# Evaluating and prioritizing the healthcare waste disposal center locations using a hybrid multi-criteria decision-making method

**DOI:** 10.1038/s41598-023-42455-w

**Published:** 2023-09-13

**Authors:** Mohammad Ali Beheshtinia, Fatemeh Bahrami, Masood Fathi, Shahla Asadi

**Affiliations:** 1 Industrial Engineering Department, Faculty of Engineering, Semnan University, Semnan, Iran; 2 Division of Intelligent Production Systems, School of Engineering Science, University of Skövde, 54128 Skövde, Sweden; 3 Division of Industrial Engineering and Management, Department of Civil and Industrial Engineering, Uppsala University, 75121 Uppsala, Sweden; 4 Department of Information Systems and Business Analytics, Kent State University, Kent, OH 44242 USA

**Keywords:** Environmental sciences, Health care

## Abstract

Healthcare waste disposal center location (HCWDCL) impacts the environment and the health of living beings. Different and sometimes contradictory criteria in determining the appropriate site location for disposing of healthcare waste (HCW) complicate the decision-making process. This research presents a hybrid multi-criteria decision-making (MCDM) method, named PROMSIS, to determine the appropriate HCWDCL in a real case. The PROMSIS is the combination of two well-known MCDM methods, namely TOPSIS and PROMETHEE. Moreover, fuzzy theory is used to describe the uncertainties of the problem parameters. To provide a reliable decision on selecting the best HCWDCL, a comprehensive list of criteria is identified through a literature review and experts’ opinions obtained from the case study. In total, 40 criteria are identified and classified into five major criteria, namely economic, environmental, social, technical, and geological. The weight of the considered criteria is determined by the Analytical Hierarchy Process (AHP) method. Then, the score of the alternative HCWDCLs in each considered criterion is obtained. Finally, the candidate locations for disposing of HCWs are ranked by the proposed fuzzy PROMSIS method. The results show that the most important criteria in ranking the alternatives in the studied case are economic, environmental, and social, respectively. Moreover, the sub-criteria of operating cost, transportation cost, and pollution are identified as the most important sub-criteria, respectively.

## Introduction

The healthcare waste disposal center location (HWDCL) problem has always been an important concern for practitioners due to its impact on society and the environment^1^. Nowadays, the increase in population and the cities’ expansions caused an increase in the need for healthcare centers such as general and specialized hospitals, clinics and polyclinics, maternity hospitals, etc.^2^.

With the increase in the number of healthcare centers, both the amount and variety of Healthcare Waste (HW) have significantly grown. According to the report from Statista^3^, the volume of HW in 2018 was substantial across various countries. For example, India produces 550 metric tons of biomedical waste daily^3^. Moreover, according to the report by the united states association, hospitals generate approximately 5.9 million tons of various types of HCW in a year^4^. Moreover, unforeseen events like the COVID-19 pandemic have further increased the volume of healthcare waste worldwide. Statista reports that in Bangkok, the volume of healthcare waste grew from 26 metric tons per day pre-crisis to 160 metric tons per day during the crisis^5^. Statista also highlighted that approximately 15% percent of this waste is categorized as hazardous, which includes infectious, toxic, or radioactive waste. Studies show that 2.5 million people, most of them children, die each year from HCW-related diseases^6^. These facts and figures collectively emphasize the importance of suitable and effective disposal management practices.

The main sources of creating HCW are hospitals and other health facilities, laboratories and research centers, nursing homes for the elderly, mortuary and autopsy centers, and animal research and testing laboratories. HCWs have various types, such as pathological waste, sharps waste, chemical waste, pharmaceutical waste, cytotoxic waste, and radioactive waste^7^. These types of HCWs may cause dangerous threats to public health. For example, a person who experiences one needle stick injury from a needle used on an infected source patient has risks of 30%, 1.8%, and 0.3%, respectively, of becoming infected with HBV, HCV, and HIV^8^. In such circumstances, the HCWs should not be mixed with municipal wastes because the dispersion of various chemical and biological materials causes hazardous and dangerous pollution that affects society’s health^9^. Therefore, HCW must be separately disposed of in certain locations ensuring the minimum impact on the environment and health of living beings.

A report by Statista shows that only a little over half the countries in the world have any legislation regarding HCW management as of 2020^10^. This reveals the lack of legal frameworks to effectively manage HCWs, thereby increasing the risk of improper disposal. The improper HWDCL increases environmental pollution, possibly leading to many social and environmental problems^11^.

One of the most important concerns in waste management is determining the best place for the HCWs disposal center due to their highly hazardous effects. HCWDCL is a critical strategic problem faced by healthcare specialists and municipalities^2^. In the real world, various aspects such as environmental, social, economic, geographical, and technical must be taken into consideration in the HWDCL problem^12^.

The existence of several criteria that may be incompatible or dependent on each other makes it challenging to determine the appropriate site location for the HCW disposal centers. In such circumstances, Multi-Criteria Decision Making (MCDM) methods are often used to decide on the best location for HCW disposal centers. The MCDM methods help decision-makers select the most desirable choice among several alternatives considering a set of criteria^13^.

Considering various and comprehensive criteria in determining the best location for HCW disposal means taking various aspects into consideration in the decision-making process, which consequently increases the reliability of results. This research proposes a hybrid MCDM method, named PROMSIS, for the HWDCL problem and applies it to a real case. The proposed PROMSIS method is a combination of the TOPSIS and PROMETHEE methods that integrates the viewpoints of these two methods to have a comprehensive viewpoint in ranking the alternatives. Moreover, fuzzy theory is employed to describe the uncertainties of the problem parameters.

In selecting the best HCWDCL in the case study, a new and comprehensive set of criteria composed of 5 main criteria (i.e., considering economic, environmental, social, technical, and geological) and 40 sub-criteria are considered. The related criteria are identified according to the literature and experts’ opinions. Then, the weight of the selected criteria is calculated using the Analytical Hierarchy Process (AHP) method. After determining some candidate sites as alternatives, the score of each alternative in each criterion is determined. Finally, the ranking of HCWDCLs is determined using the fuzzy PROMSIS method.

The main innovation of this research is considering a comprehensive list of sub-criteria for the HCWDCL problem, in which five of these sub-criteria are not considered by the previous research. These sub-criteria are the “distance from power lines”, “burial fee”, “project construction cost”, “the emission rate of bad odor”, and “lack of leachate control”. Another innovation of this research is introducing a new hybrid fuzzy MCDM method named PROMSIS, which is a combination of TOPSIS and PROMETHEE methods.

Considering the above explanation, the main research question of this study is as follows.

To answer the main question, the following sub-questions should be addressed.

- How can the HCWDCLs be prioritized in the studied case considering the economic, environmental, technical, and social criteria?
- What criteria should be considered for the evaluation of HCWDCLs?
- What is the importance (weight) of each identified criterion?
- What are the candidate locations (alternatives) for establishing the HCW disposal center?
- What is the score of each alternative in each considered criterion?
- What is the ranking of the alternative HCWDCLs using the PROMSIS method?

The remainder of the paper is organized as follows. Section “Literature review” presents a review of the literature on the HWDCL problem. The research methodology is presented in section “Methodology”. The results of the implementation of the research steps are presented in section “Implementation, results, and discussion”. Finally, the conclusion and areas for future research are explained in section “Conclusion and scopes of future research”.

## Literature review

Various researchers have used the MCDM methods in the scope of waste management. Haseli and Jafarzadeh Ghoushchi^14^ used the base-criterion method (BCM) and combined it with spherical fuzzy sets (SFSs) to evaluate and determine the locations of waste disposal in the city of Tabriz, Iran. Zafaranlouei et al.^15^ merged BCM and combined compromise solution (CoCoSo) methods under fuzzy Z-numbers to rank 21 types of waste based on economic, social, and environmental criteria, as well as 13 sub-criteria related to those criteria. Haseli, et al.^16^ proposed an integrated approach using Z-numbers based on the best–worst method (BWM) and the CoCoSo to solve the recycling partner selection problem.

The waste management problem has been extensively studied in the literature, and several solutions have been proposed to address it. This section only reviews the most recent studies HWDCL problem where an MCDM method has been employed. The review aims to identify the previously considered criteria and use MCDM methods for selecting HCWDCL.

Chauhan and Singh^17^ employed a hybrid MCDM method, including Fuzzy AHP and Fuzzy 'technique for order preference and similarity to the ideal solution’ (TOPSIS), to select a sustainable location for the HWDCL problem in Garhwal, India. The environmental, social, and economic criteria and eight sub-criteria were considered for ranking the seven candidate centers. Wichapa and Khokhajaikiat^7^ discussed the HWDCL problem in the sub-Northeast region of Thailand, considering 47 hospitals and three municipalities. They used a combination of Fuzzy AHP and goal programming (GP) methods to solve the problem considering infrastructure, geological, environmental, and social criteria. Wichapa and Khokhajaikiat^18^ used a hybrid MCDM method, including Fuzzy AHP and Fuzzy TOPSIS, to find the best HCW disposal site to gather wastes from 40 hospitals in the northeastern region of Thailand. They considered criteria such as infrastructure, geological, environmental, and social factors in the considered HCW disposal center location problem.

Mohammed et al.^19^ employed the AHP method by integrating dynamic data such as future population and forecasting waste generation to provide suitable locations for the HCW disposal center in Johor Bahru, Malaysia. In this research, 13 criteria of water bodies, soil, geology, slope, elevation, residential areas, archaeological sites, airports, population, road, railway, infrastructure, and land use were considered. Stemn and Kumi-Boateng^20^ studied the selection of hazardous waste disposal sites in the Western Region of Ghana. Four main criteria (i.e., geographic, environmental, economic, and social) and 32 sub-criteria were considered for this study. Finally, using the AHP method, waste disposal sites were ranked. Yazdani et al.^4^ studied the location of an HCW disposal center in Madrid, Spain. For this purpose, three main criteria (i.e., economic, environmental, and social) and 11 sub-criteria were identified and evaluated. Finally, using three methods of Best–worst method (BWM), Combined Compromise Solution (CoCoSo), and Dombi-Bonferroni (D-B), the best place for an HCW disposal center is identified.

Ali et al.^21^ employed AHP and fuzzy TOPSIS (FTOPSIS) to choose the best HCW disposal site in Memari, India. The main criteria considered in this research were environmental and socio-economic aspects. TAŞ^22^ addressed the HWDCL problem in Turkey during the COVID-19 pandemic. They considered several criteria in this study for ranking HCW disposal centers, such as underground water, accessibility to main roads, capacity, distance to residential areas, potential adjacent land use, distance to forests, and slope. The candidate places for the HCW disposal center were ranked using the Pivot Pairwise Relative Criteria Importance Assessment (PIPRECIA) method. Ghoushchi et al.^23^ proposed the Stepwise Weight Assessment Ratio Analysis (SWARA) and the Weighted Aggregated Sum Product Assessment (WASPAS) methods based on spherical fuzzy sets (SFS) to select the best HCW disposal site in Urmia, Iran. They identified criteria for choosing the best HCW disposal site, including three main criteria (i.e., environmental, economic, and social) and 13 sub-criteria. The weight of 13 sub-criteria was calculated by Spherical Fuzzy Step-Wise Weight Assessment Ratio Analysis (SFSWARA). The candidate sites for an HCW disposal center were evaluated and ranked using Spherical Fuzzy Weighted Aggregated Sum Product Assessment (SFWASPAS). Mishra and Rani^2^ discussed selecting the best HCW disposal site in Uttarakhand, India. For this purpose, three main criteria (i.e., economic, environmental, and social) and ten sub-criteria were identified and evaluated. Finally, Fermatean Fuzzy WASPAS (FF-WASPAS) method was applied to rank the alternative sites for the HCW waste disposal center. Salimian and Mousavi^24^ proposed the method of ordered weight averaging (OWA) to solve the HWDCL problem in Uttarakhand, India. This study identified and evaluated three main criteria (Environmental, Economic, Social) and ten sub-criteria. Torkayesh, et al.^11^ used the combination of the Best–Worst Method (BWM) and 'Measurement of alternatives and ranking according to compromise solution' (MARCOS) to locate an HCW disposal center in Hamedan, Iran. In this study, eight candidate sites were considered alternatives, and four main criteria of geology, technical, environmental, and economical, and 16 sub-criteria were identified to evaluate the alternatives. Tirkolaee and Torkayesh^25^ presented three methods (Stratified Best–Worst Method (SBWM), MARCOS, and CoCoSo) for solving the HWDCL problem in Mazandaran, Iran considering 79 medical centers. They used three main criteria (Social, Economic, and Environmental) and nine sub-criteria to rank the candidate sites.

Simic et al.^13^ tried to find a good location for the HCW disposal site in Istanbul, Turkey. For this purpose, five candidate sites were evaluated by four main criteria, namely social, technical, economic, and environmental. Finally, the best place was identified using Indifference Threshold-based Attribute Ratio Analysis (ITARA), random forest recursive feature elimination (RF-RFE), and measurement of alternatives and ranking according to compromise solution (MARCOS) methods. Ghoushchi and Nasiri^26^ investigated the location of the HCW disposal site. They considered three main criteria (i.e., environmental, social, and economic) and 13 sub-criteria. The authors applied the SWARA and G-number methods to find the best site for the HCW disposal center. Torkayesh and Simic^27^ used combinations of the Hierarchical Stratified Best–Worst Method (H-SBWM), CoCoSo, and WASPAS methods to determine the best site for recycling facility for urban healthcare plastic wastes in Istanbul, Turkey. They considered four main criteria (technical, economic, environmental, and social) and 16 sub-criteria to evaluate the candidate sites.

The literature review shows that several criteria and various MCDM methods are considered to evaluate and prioritize the HCWDCL. Table 1 summarizes the criteria and MCDM methods used in the reviewed studies.

**Table 1:** The considered criteria and used MCDM approach in the reviewed studies.

Research	Criteria																							
	Economic								Environmental								Social							
	Operating costs	Transport cost	The cost of repairs and maintenance	Investment cost	Land price	Burial fee	Possibility of future expansion	Project construction cost	Contamination (air, water, soil)	Distance from surface water	Noise pollution	The effect of greenhouse gas emissions	Depth of groundwater	Distance from residential areas	Odor emission rate	Distance from power lines	Social acceptance	Population density	Archeological importance	Distance from communities	Health & safety	Adherence to rules and regulations	Land-use	Lack of municipal problems
Chauhan and Singh^17^		*							*			*											*	
Wichapa and Khokhajaikiat^7^		*	*		*					*								*		*				*
Wichapa and Khokhajaikiat^18^										*								*		*				*
Mohammed, et al.^19^																		*	*				*	
Stemn and Kumi-Boateng^20^					*					*			*						*					
Yazdani, et al.^4^		*	*		*		*		*													*		
Ali, et al.^21^					*					*				*				*		*			*	
TAŞ^22^							*						*	*										
Ghoushchi, et al.^23^										*			*				*			*			*	
Mishra and Rani^2^		*	*		*		*															*		
Salimian and Mousavi^24^			*		*		*													*		*		
Torkayesh, et al.^11^					*		*						*							*		*	*	
Tirkolaee and Torkayesh^25^		*	*		*		*		*					*								*		
Simic, et al.^13^	*	*	*	*	*				*		*										*	*		
Ghoushchi and Nasiri^26^				*						*			*										*	
Torkayesh and Simic^27^			*	*			*				*	*					*					*		
Current research	*	*	*	*	*	*	*	*	*	*	*	*	*	*	*	*	*	*	*	*	*	*	*	*

Research	Criteria																Applied techniques							
	Technical								Geological															
	Landfill capacity	Availability of workforce	Accessibility to road	Infrastructure development	Distance to the garbage collection point	Distance to a complex of waste sorting	Distance from mines	Lack of leachate control	Soil type	Height	Distance from the fault	Slope	Wind problems	Flooding	Precipitation estimates	Vegetation								
Chauhan and Singh^17^	*		*		*												AHP TOPSIS							
Wichapa and Khokhajaikiat^7^														*			AHP GP							
Wichapa and Khokhajaikiat^18^														*			AHP TOPSIS							
Mohammed, et al.^19^				*					*	*		*					AHP							
Stemn and Kumi-Boateng^20^							*		*		*	*	*			*	MSW							
Yazdani, et al.^4^						*											BWM, CoCoSo, D-B							
Ali, et al.^21^										*						*	AHP, FTOPSIS							
TAŞ^22^			*	*													PIPRECIA							
Ghoushchi, et al.^23^											*	*			*		SWARA, WASPAS							
Mishra and Rani^2^		*				*											FF-WASPAS							
Salimian and Mousavi^24^		*															OWA-IF							
Torkayesh, et al.^11^									*		*	*	*		*		BWM MARCOS							
Tirkolaee and Torkayesh^25^						*											MARCOS-CoCoSo SBWM							
Simic, et al.^13^		*				*											ITARA, MARCOS, RF-RFE							
Ghoushchi and Nasiri^26^												*				*	SWARA, G-number							
Torkayesh and Simic^27^					*												H-SBWM CoCoSo WASPAS							
Current research	*	*	*	*	*	*	*	*	*	*	*	*	*	*	*	*	AHP PROMSIS							

On the bases of the performed review, the contribution and novelty of the current study can be stated as follows:

- Considering a comprehensive list of sub-criteria, including 40 sub-criteria divided into five main criteria (i.e., economic, environmental, social, technical, and geological), to select the best HCW disposal site. Considering a comprehensive list of criteria and sub-criteria means making a more reliable decision by paying attention to different aspects when selecting the HCWDCL.
- Introducing five new sub-criteria motivated by the case study, which are not considered by the previous researches. These sub-criteria are the “Distance from power lines”, “Burial fee”, and “Project construction cost”, “The emission rate of bad odor”, and “Lack of leachate control”.
- Introducing a hybrid fuzzy MCDM method (i.e., PROMSIS), which is a combination of TOPSIS and PROMETHEE methods.

## Methodology

This study deals with evaluating and prioritizing HCWDCLs considering economic, social, environmental, technical, and geological criteria. A hybrid MCDM method named PROMSIS is introduced to tackle the problem. Moreover, fuzzy theory is used to describe the problem parameters’ uncertainty. In this research, fuzzy triangular numbers are used to determine the values of linguistic terms. Each triangular number $\tilde{A}$ = (*l,m,u*) is proposed by three elements of *l, m,* and *u* that show the lowest, most likely, and highest value for the number, respectively. The used mathematical calculation between two fuzzy numbers of $\tilde{L} = \left( {l_{1} ,m_{1} ,u_{1} } \right)$ and $\tilde{M} = \left( {l_{2} ,m_{2} ,u_{2} } \right)$ are presented in Eqs. (1, 2, 3, 4, 5)^28,29^.

1 $$\tilde{L} + \tilde{M} = \left( {l_{1} + l_{2} ,m_{1} + m_{2} ,u_{1} + u_{2} } \right)$$

2 $$\tilde{L} - \tilde{M} = \left( {l_{1} - u_{2} ,m_{1} - m_{2} ,u_{1} - l_{2} } \right)$$

3 $$\tilde{L} \times \tilde{M} = \left( {\min \left( {l_{1} l_{2} ,l_{1} u_{2} ,l_{2} u_{1} ,u_{1} u_{2} } \right),m_{1} m_{2} ,\max \left( {l_{1} l_{2} ,l_{1} u_{2} ,l_{2} u_{1} ,u_{1} u_{2} } \right)} \right)$$

4 $$\tilde{L}/\tilde{M} = \left( {\min \left( {l_{1} /l_{2} ,l_{1} /u_{2} ,u_{1} /l_{2} ,u_{1} /u_{2} } \right),m_{1} /m_{2} ,\max \left( {l_{1} /l_{2} ,l_{1} /u_{2} ,u_{1} /l_{2} ,u_{1} /u_{2} } \right)} \right)$$

5 $${\text{Distance}} (\tilde{L},\tilde{M}) = \sqrt {\frac{1}{3} \times \left\{ {(l_{1} - l_{2} )^{2} + (m_{1} - m_{2} )^{2} + (u_{1} - u_{2} )^{2} } \right\}}$$

Moreover, defuzzification of fuzzy number $\tilde{A}$ = (*l,m,u*) is obtained using Eq. (6).

6 $${\text{Defuzzify}} \;\left( {\tilde{A}} \right) = \frac{l + 4m + u}{6}$$

### Research steps

The following five steps are taken to evaluate and prioritize the HCWDCLs in this study. It is worth mentioning this study benefits from two questionnaires. The first questionnaire is a pairwise comparisons matrix (used in the AHP method). In the pairwise comparisons matrix questionnaire, each respondent uses a number between 1 (Just equal) to 9 (Extremely Preferred) to determine the privilege of two criteria against each other.

The second is a decision matrix questionnaire. In the decision matrix questionnaire, each respondent uses the Likert scale to determine the score of each candidate site (alternative) in each criterion. The linguistic terms used in the Likert scale and their corresponding fuzzy numbers are as follows: (1) Very low with the value of (0, 0, 0.75), (2) Low with the value of (0.5, 1.25, 2), (3) Average with the value of (1.75, 2.5, 3.25), (4) High with the value of (3, 3.75, 4.5), and (5) Very high with the value of (4.25, 5, 5).

The questionnaires used in this study are standard, and their validity is confirmed by previous studies^21,30^. Both questionnaires are filled out by ten experts. Table 2 shows the experts’ information.

**Table 2:** Experts’ information.

Job	Academic degree	Experiences (years)	Expertise
Hospital manager	Ph.D.	12	Doctor of medicine
Clinic manager	Ph.D.	15	Doctor of medicine
Clinic manager	Ph.D.	13	Doctor of medicine
Municipal recycling manager	M.Sc.	15	Civil engineering
Municipal recycling manager	B.Sc.	18	Civil engineering
Municipal green space manager	M.Sc.	18	Civil engineering
Environment health manager in a recycling company	M.Sc.	21	Environment health
Environment health manager at the public health department	M.Sc.	20	Environment health
Logistic manager in a recycling company	M.Sc.	14	Industrial engineer
Logistic manager in a recycling company	B.Sc.	18	Industrial engineer

***Step 1.*** Determine the effective criteria and sub-criteria in evaluating and prioritizing HCWDCLs. These criteria are obtained by reviewing the literature and considering experts' opinions.

***Step 2.*** Determine the weight of the considered criteria and sub-criteria using the AHP method. A pairwise comparison questionnaire is used to obtain the input matrix needed for the AHP method^31^. This questionnaire compares the effective criteria for deciding on the HCWDCL. In comparing the two criteria, the respondents were asked to choose one of the following alternatives: (1) Very slightly preferred, (2) Slightly preferred, (3) Preferred, (4) Preferred, and (5) Very highly preferred^32^. These linguistic terms are converted to a number to obtain the criteria weights. The considered values for the first to fifth linguistic terms are 1, 3, 5, 7, and 9, respectively .

A pairwise comparisons matrix is formed as presented in Eq. (7), where *n* and* a*_*kj*_ are the numbers of criteria, and the privilege of criterion *k* against criterion *j*, respectively^33^.

7 $${\text{A}} = \left[ {a_{kj} } \right]_{n \times n} = \left[ {\begin{array}{*{20}c} {a_{11} } & {a_{12} } & {a_{13} } & \ldots & {a_{1n} } \\ {a_{21} } & {a_{22} } & {a_{23} } & \ldots & {a_{12} } \\ {a_{31} } & {a_{32} } & {a_{33} } & \ldots & {a_{13} } \\ \ldots & \ldots & \ldots & \cdots & \ldots \\ \ldots & \ldots & \ldots & \ldots & \ldots \\ {a_{n1} } & {a_{n2} } & {a_{n3} } & \cdots & {a_{nn} } \\ \end{array} } \right] \;\;\;\; k, j = 1, 2, \ldots , n$$

Then, the pairwise comparisons matrix is normalized using Eq. (8), where *H*_*kj*_ is the normalized value of *a*_*kj*_.

8 $$H_{kj} = {{a_{kj} } \mathord{\left/ {\vphantom {{a_{kj} } {\mathop \sum \limits_{k = 1}^{n} a_{kj} }}} \right. \kern-0pt} {\mathop \sum \limits_{k = 1}^{n} a_{kj} }}\;\; \;k, j = 1, 2, \ldots , n$$

The final weight of each criterion is obtained by Eq. (9), where *w*_*j*_ is the final weight of criterion *i*.

9 $$w_{j} = \mathop \sum \limits_{k = 1}^{n} \frac{{H_{kj} }}{n}\;\;\;\;j = 1, 2, \ldots , n$$

A pairwise comparison matrix should be established to compare the main criteria. Moreover, a pairwise comparisons matrix should be established for each main criterion to compare its related sub-criteria. The final weight of each sub-criterion is calculated by multiplying its weight by the weight of its corresponding main criterion.

**S*tep 3.*** Determine candidate locations (alternative) for the HCWDCL. Experts usually determine the alternatives based on some factors.

***Step 4.*** Create the decision matrix (determine the score of each candidate for the HCWDCL in each criterion) using the decision matrix questionnaire. This questionnaire is used to determine the score of each HCW disposal candidate site in each of the considered criteria and form the decision matrix.

***Step 5.*** Rank the alternatives using the fuzzy PROMSIS method. Fuzzy PROMSIS is a combination of fuzzy TOPSIS and fuzzy PROMETHEE methods. The details of the fuzzy PROMSIS are described in section “The proposed fuzzy PROMSIS”.

### The proposed fuzzy PROMSIS

In this section, the proposed fuzzy PROMSIS method is presented. As stated before, PROMSIS is a product of hybridizing TOPSIS and PROMETHEE. Each MCDM method has a different viewpoint in prioritizing the alternatives, resulting in a different solution. In the TOPSIS method, an alternative is preferable if its distance from the positive ideal solution (PIS) is low and from the negative ideal solution (NIS) is high. On the other hand, in the PROMETHEE method, an alternative is preferable if its net preference flow is high. The proposed PROMSIS method tries to integrate these two viewpoints.

In the PROMSIS method, an alternative is preferable if its distance from NIS is high, its distance from PIS is low, and its net preference flow value is simultaneously high. Having various viewpoints in ranking the alternatives caused the decision-making process to have a comprehensive attitude and reliable results. Before presenting the steps of the fuzzy PROMSIS method, the PROMETHEE and TOPSIS methods are briefly described.

#### PROMETHEE

The PROMETHEE method is an MCDM method that is popular for its simplicity, clarity, and reliability of the results^34^. This method is suitable for evaluating a limited set of alternatives in the form of a partial or complete ranking.

This method gives the decision matrix (i.e., the score of each alternative in each criterion) and the weight of the criteria as its input. Suppose the decision matrix is as presented in Eq. (10), the main steps of the PROMETHEE method can be explained as follows.

10 $$X = \left[ {x_{ij} } \right]_{m \times n} = \left[ {\begin{array}{*{20}c} {x_{11} } & {x_{12} } & {x_{13} } & \ldots & {x_{1n} } \\ {x_{21} } & {x_{22} } & {x_{23} } & \ldots & {x_{2n} } \\ {x_{31} } & {x_{32} } & {x_{33} } & \ldots & {x_{3n} } \\ \ldots & \ldots & \ldots & \cdots & \ldots \\ \ldots & \ldots & \ldots & \ldots & \ldots \\ {x_{m1} } & {x_{m2} } & {x_{m3} } & \cdots & {x_{mn} } \\ \end{array} } \right] \;\;\;i = 1, 2, \ldots , m;\;\; j = 1, 2, \ldots , n$$

where $x_{ij}$, *n* and *m* are the scores of alternative *i* in criterion *j*, number of criteria, and number of alternatives, respectively.

***Step 1*.** Calculate the difference of alternatives in different criteria with pairwise comparisons of alternatives in each criterion using Eq. (11).

11 $$d_{j} \left( {A,B} \right) = x_{Aj} - x_{Bj}$$

where $d_{j} \left( {A,B} \right)$ is the difference between the score of alternative *A* against alternative *B* in criterion *j*. This difference represents the privilege of alternative *A* against *B* if $x_{Aj} \ge x_{Bj}$ for the profit criteria, or $x_{Aj} \le x_{Bj}$ for the cost criteria.

***Step 2*.** Calculate the superiority of the alternatives against each other according to the superiority function *P*. There are six superiority functions, each of which takes the value of $d_{j} \left( {A,B} \right)$ as the input and gives a value between 0 and 1 as the output^35^. In this research, a Gaussian preference function is used, as shown in Eq. (12).

12 $$P_{j} (A,B) = P\left[ {d_{j} (A,B)} \right] = \left\{ {\begin{array}{*{20}l} {1 - e^{{\frac{{ - (d_{j} (A,B))^{2} }}{{2\sigma_{j}^{2} }}}} } \hfill & {if\,d_{j} (A,B) > 0} \hfill \\ 0 \hfill & {if\,d_{j} (A,B) \le 0} \hfill \\ \end{array} } \right.$$

The value of $\sigma$ represents the threshold value between the indifferent and strict preference areas. In the other preference functions, if $d_{j} \left( {A,B} \right)$ is bigger than a threshold value, the function returns value of 1. In this case, the differences between $d_{j} \left( {A,B} \right)$ values are neglected. For example, if the threshold *p* is equal to 1, then there is no difference between $d_{j} \left( {A,B} \right) = 1$ and $d_{j} \left( {A,B} \right) = 5$. But the Gaussian method considers any differences between $d_{j} \left( {A,B} \right)$ values.

***Step 3.*** Calculate the multi-criteria preference degree of alternative *A* against alternative *B* using Eq. (13).

13 $${\uppi }\left( {A,B} \right) = \mathop \sum \limits_{{}} P_{j} \left( {A,B} \right) \times w_{j}$$

***Step 4*.** Calculate the input preference flow (*Φ*^*in*^) and the output preference flow (*Φ*^*out*^) of each alternative using Eqs. (14) and (15). The input preference flow indicates how much an alternative like *A* is superior to other alternatives. The higher this value is, the better this alternative is. The output preference flow indicates how much other alternatives are superior to alternative *A*. The lower this value is, the better this alternative is.

14 $${\Phi }^{in} \left( A \right) = \frac{{\mathop \sum \nolimits_{x = 1}^{m} \pi \left( {A,x} \right)}}{{\left( {n - 1} \right)}}$$

15 $${\Phi }^{out} \left( A \right) = \frac{{\mathop \sum \nolimits_{x = 1}^{m} \pi \left( {x,A} \right)}}{{\left( {n - 1} \right)}}$$

***Step 5*.** Calculate the net preference flow (*Φ*) for each alternative using Eq. (16). The higher the net preference flow of an alternative, the better it is.

16 $$\Phi_{{\text{A}}} = \Phi^{in} \left( A \right){-} \Phi^{out} \left( A \right)$$

#### TOPSIS

The TOPSIS is a well-known MCDM method. The TOPSIS method identifies the PIS and NIS. This method prefers an alternative whose sum of its distance from PIS is low and, simultaneously, its distance from NIS is high^36^. The main steps of the TOPSIS are summarized below.

***Step 1.*** Calculate the normalized decision matrix *R* = [*r*_*ij*_]_m×n_ using Eq. (17). Where ${x}_{ij}$ is the score of alternative *i* in criterion *j*.

17 $$r_{ij } = \frac{{x_{ij} }}{{\sqrt {\mathop \sum \nolimits_{i = 1}^{m} x_{ij}^{2} } }}$$

***Step 2.*** Obtain the weighted normalized decision matrix *V* = [*v*_*ij*_]_*m*×*n*_ using Eq. (18).

18 $$v_{ij} = w_{j} *r_{ij}$$

***Step 3.*** Calculate $PIS = \left[ {pis_{1}^{{}} ,pis_{2}^{{}} , \cdots ,pis_{n}^{{}} } \right]$ and $NIS = \left[ {nis_{1}^{{}} ,nis_{2}^{{}} , \cdots ,nis_{n}^{{}} } \right]$ using Eqs. (19) and (20). Where *J*^+^ indicates the set of profit criteria, and *J*^*−*^ indicates the set of cost criteria.

19 $$pis_{i}^{{}} = \left\{ {\begin{array}{*{20}c} {\max v_{ij} \,\,\,\,\,\,if\,j \in J^{ + } } \\ {\min v_{ij} \,\,\,\,\,if\,j \in J^{ - } } \\ \end{array} } \right.\,\,\,\,\,\,\,\forall i = 1,2,...,m$$

20 $$nis_{i}^{{}} = \left\{ {\begin{array}{*{20}c} {\min v_{ij} \,\,\,\,\,\,if\,j \in J^{ + } } \\ {\max v_{ij} \,\,\,\,\,if\,j \in J^{ - } } \\ \end{array} } \right.\,\,\,\,\,\,\,\forall i = 1,2,...,m$$

***Step 4.*** Calculate the distance of each alternative from the PIS ($DPIS_{i}$) and the NIS ($DNIS_{i}$) using Eqs. (21) and (22).

21 $$DPIS_{i} = \sqrt {\sum\limits_{j = 1}^{n} {\left( {\mathop v\nolimits_{ij} - \mathop {pis}\nolimits_{j}^{{}} } \right)^{2} } }$$

22 $$DNIS_{i} = \sqrt {\sum\limits_{j = 1}^{n} {\left( {\mathop v\nolimits_{ij} - \mathop {nis}\nolimits_{j}^{{}} } \right)^{2} } }$$

***Step 5.*** Calculate the closeness coefficient for each alternative (*CC*_*i*_) using Eq. (23).

23 $$CC_{i} = \frac{{DNIS_{i} }}{{DPIS_{i} + DNIS_{i} }}$$

***Step 6.*** Sort the alternatives in descending order of closeness coefficient. Consider the alternative that has the highest value of closeness coefficient as the best alternative.

#### Fuzzy PROMSIS

The PROMSIS method and its steps are described in this section. In the fuzzy PROMSIS method, the decision matrix (i.e., the score of each alternative in each criterion) and the weight of the criteria are taken as input. Equation (24) shows the fuzzy decision matrix.

24 $$\begin{aligned} \tilde{X} & = \left[ {\tilde{x}_{ij} } \right]_{m \times n} = \left[ {\begin{array}{*{20}c} {\tilde{x}_{11} } & \cdots & {\tilde{x}_{1n} } \\ \vdots & \ddots & \vdots \\ {\tilde{x}_{m1} } & \cdots & {\tilde{x}_{mn} } \\ \end{array} } \right] = \left[ {\begin{array}{*{20}c} {\left( {x_{11}^{l} ,x_{11}^{m} ,x_{11}^{u} } \right)} & \cdots & {\left( {x_{1n}^{l} ,x_{1n}^{m} ,x_{1n}^{u} } \right)} \\ \vdots & \ddots & \vdots \\ {\left( {x_{m1}^{l} ,x_{m1}^{m} ,x_{m1}^{u} } \right)} & \cdots & {\left( {x_{mn}^{l} ,x_{mn}^{m} ,x_{mn}^{u} } \right)} \\ \end{array} } \right]\; \\ i & = 1, 2, \ldots , m;j = 1, 2, \ldots , n \\ \end{aligned}$$

where $\tilde{x}_{ij}$, *n* and *m* are the scores of alternative *i* in criterion *j*, number of criteria, and number of alternatives, respectively.

The steps of the fuzzy PROMSIS method are as follows.

***Step 1.*** Calculate the difference of alternatives in different criteria with pairwise comparisons of alternatives in each criterion. Equation (25) is used for profit criteria, and Eq. (26) for cost criteria. Where $d_{j} \left( {A,B} \right)$ is the difference between alternatives A and B in criterion *j*. This difference represents the privilege of alternative *A* against *B*. In this research, the fuzzy distance of these two numbers is obtained by Eq. (5).

25 $$\begin{gathered} d_{j} (A,B) = {\text{Distance}} \left( {\tilde{x}_{Aj} ,\tilde{x}_{Bj} } \right) = \left\{ {\begin{array}{*{20}c} {\sqrt {\frac{1}{3} \times \left\{ {\left( {x_{Aj}^{l} - x_{Bj}^{l} } \right)^{2} + \left( {x_{Aj}^{m} - x_{Bj}^{m} } \right)^{2} + \left( {x_{Aj}^{u} - x_{Bj}^{u} } \right)^{2} } \right\}} } & {if\,\tilde{x}_{Aj} > \tilde{x}_{Bj} \,\,} \\ 0 & {otherwise} \\ \end{array} } \right. \hfill \\ \hfill \\ \end{gathered}$$

26 $$d_{j} (A,B) = {\text{Distance}} \left( {\tilde{x}_{Aj} ,\tilde{x}_{Bj} } \right) = \left\{ {\begin{array}{*{20}c} {\sqrt {\frac{1}{3} \times \left\{ {\left( {x_{Aj}^{l} - x_{Bj}^{l} } \right)^{2} + \left( {x_{Aj}^{m} - x_{Bj}^{m} } \right)^{2} + \left( {x_{Aj}^{u} - x_{Bj}^{u} } \right)^{2} } \right\}} } & {if\,\,\tilde{x}_{Aj} < \tilde{x}_{Bj} \,} \\ 0 & {otherwise} \\ \end{array} } \right.$$

where $\tilde{x}_{Aj} = \left( {x_{Aj}^{l} ,x_{Aj}^{m} ,x_{Aj}^{u} } \right)$ and $\tilde{x}_{Bj} = \left( {x_{Bj}^{l} ,x_{Bj}^{m} ,x_{Bj}^{u} } \right)$ are the score of alternatives A and B in criterion *j*, respectively.

***Step 2.*** Calculate the superiority of the alternatives over each other in each criterion according to the superiority function using Eq. (27). In this research, the GAUSSIAN superiority function is used with $\sigma =0.5$.

27 $$P_{j} (A,B) = P(d_{j} (A,B)) = 1 - e^{{\frac{{ - (d_{j} (A,B))^{2} }}{{2\sigma_{j}^{2} }}}} \,$$

***Step 3.*** Calculate the multi-criteria preference degree of alternative *A* against alternative *B* using Eq. (28).

28 $${\uppi }\left( {A,B} \right) = \mathop \sum \limits_{{}} P_{j} \left( {A,B} \right) \times w_{j}$$

***Step 4.*** For each alternative *A*, calculate each alternative's input preference flow (${\Phi }^{in}\left(A\right)$), output preference flow (${\Phi }^{out}\left(A\right)$) and net preference flow (*Φ*_*A*_) using Eqs. (29, 30, 31), respectively.

29 $${\Phi }^{in} \left( A \right) = \frac{{\mathop \sum \nolimits_{x = 1}^{m} \pi \left( {A,x} \right)}}{{\left( {n - 1} \right)}}$$

30 $${\Phi }^{out} \left( A \right) = \frac{{\mathop \sum \nolimits_{x = 1}^{m} \pi \left( {x,A} \right)}}{{\left( {n - 1} \right)}}$$

31 $$\Phi_{A} = \Phi^{in} \left( A \right) \, {-} \Phi^{out} \left( A \right)$$

***Step 5.*** Calculate the normalized decision matrix $\tilde{R} = [\tilde{r}_{ij} ]_{m \times n}$ using Eq. (32).

32 $$\tilde{r}_{ij} = \left( {r_{ij}^{l} ,r_{ij}^{m} ,r_{ij}^{u} } \right) = \left\{ {\begin{array}{*{20}c} {\left( {\frac{{x_{ij}^{l} }}{{c_{j}^{*} }},\frac{{x_{ij}^{m} }}{{c_{j}^{*} }},\frac{{x_{ij}^{u} }}{{c_{j}^{*} }}} \right)} & {c_{j}^{*} = \max_{i} x_{ij}^{u} } & {For \, the \, positive \, criterion \, j} \\ {\left( {\frac{{a_{j}^{ \circ } }}{{x_{ij}^{u} }},\frac{{a_{j}^{ \circ } }}{{x_{ij}^{m} }},\frac{{a_{j}^{ \circ } }}{{x_{ij}^{l} }}} \right)} & {a_{j}^{^\circ } = \min_{i} x_{ij}^{l} } & {For \, the \, negative \, criterion \, j} \\ \end{array} } \right.$$

***Step 6.*** Obtain the weighted normalized decision matrix $\tilde{V} = [\tilde{v}_{ij} ]_{m \times n}$ using Eq. (33).

33 $$\tilde{v}_{ij} = w_{j} \times \tilde{r}_{ij} = \left( {w_{j} \times r_{ij}^{l} ,w_{j} \times r_{ij}^{m} ,w_{j} \times r_{ij}^{u} } \right)$$

***Step 7.*** Calculate Fuzzy Positive Ideal Solution (FPIS) and Fuzzy Negative Ideal Solution (FNIS) using Eqs. (34) and (35). Where *J*^+^ indicates the set of profit criteria, and *J*^*−*^ indicates the set of cost criteria $\left( {FPIS} \right. = \left[ {\widetilde{fpis}_{1} ,\widetilde{fpis}_{2} , \ldots ,\widetilde{fpis}_{n} } \right]$ and $FNIS = \left. {\left[ {\widetilde{fnis}_{1} ,\widetilde{fnis}_{2} , \ldots ,\widetilde{fnis}_{n} } \right]} \right)$.

34 $$\widetilde{fpis}_{i} = \left\{ {\begin{array}{*{20}c} {\max \tilde{v}_{ij} \,\,\,\,\,\,if\,j \in J^{ + } } \\ {\min \tilde{v}_{ij} \,\,\,\,\,if\,j \in J^{ - } } \\ \end{array} } \right.\,\,\,\,\,\,\,\forall i = 1,2,...,m$$

35 $$\widetilde{fnis}_{i} = \left\{ {\begin{array}{*{20}c} {\min \tilde{v}_{ij} \,\,\,\,\,\,if\,j \in J^{ + } } \\ {\max \tilde{v}_{ij} \,\,\,\,\,if\,j \in J^{ - } } \\ \end{array} } \right.\,\,\,\,\,\,\,\forall i = 1,2,...,m$$

***Step 8.*** Calculate the distance of each alternative *i* from the FPIS (*DPIS*_*i*_) and the FNIS (*DNIS*_*i*_) using Eqs. (36) and (37).

36 $$DNIS_{i} = \sum\limits_{j = 1}^{n} {{\text{Distance}}} \left( {\tilde{v}_{ij} ,\widetilde{fnis}_{j} } \right)\,\,\,\,\,\,\,i = 1,...,m$$

37 $$DPIS_{i} = \sum\limits_{j = 1}^{n} {\text{Distance}} \left( {\tilde{v}_{ij} ,\widetilde{fpis}_{j} } \right)\,\,\,\,\,\,\,i = 1,...,m$$

***Step 9.*** Normalize the values of *DPIS*_*i*_*, DNIS*_*i*_ and *Φ*_*i*_ using Eqs. (38) to (40).

38 $$NDNIS_{i} = \frac{{DNIS_{i} }}{{\sqrt {\sum\limits_{i = 1}^{m} {DNIS^{2}_{i} } } }}\,\,\,\,\,\,\,i = 1,...,m$$

39 $$NDNIS_{i} = \frac{{DPIS_{i} }}{{\sqrt {\sum\limits_{i = 1}^{m} {DPIS^{2}_{i} } } }}\,\,\,\,\,\,\,i = 1,...,m$$

40 $$N\Phi_{i} = \frac{{\Phi_{i} }}{{\sqrt {\sum\limits_{i = 1}^{m} {\Phi_{i}^{2} } } }}\,\,\,\,\,\,\,i = 1,...,m$$

***Step 10.*** Calculate the PROMSIS index using Eq. (41).

41 $$Q_{i} = \frac{{\left( {NDNIS_{i} + N{\Phi }_{i} } \right)}}{{NDPIS_{i} + (NDNIS_{i} + N{\Phi }_{i} )}}$$

Reviewing TOPSIS and PROMETHEE shows that the lower value of ${NDPIS}_{i}$ and the higher values of $NDNIS_{i}$ and $N{\Phi }_{i}$ are favorable in ranking the alternatives. In calculating the closeness coefficient for each alternative, its distance from PIS appears in the numerator, and the summation of its distance from PIS and NIS appears in the denominator. In the calculation of PROMSIS index, the parameters that their higher values are favorable ($NDNIS_{i}$ and $N{\Phi }_{i}$) appear in the numerator, and the summation of all the other parameters appears in the denominator.

***Step 11.*** Prioritize the alternatives according to the PROMSIS index. In this case, an alternative with a higher value of PROMSIS index gets a higher priority.

The flowchart of the research steps is presented in Fig. S1 in Supplementary Material.

## Implementation, results, and discussion

The proposed fuzzy PROMSIS has been implemented in a real case in Semnan province in Iran. This province has an area of 97,491 km^2^ and is located in geographical coordinates between 34 degrees 13 min to 37 degrees 20 min north latitude and from 51 degrees 51 min to 57 degrees 3 min east longitude. Its average height is 1130 m above sea level. This city has a population of about 702,000 persons, 41 healthcare centers, four animal research centers, and 37 healthcare laboratories that produce various types of HCW.

The province's population is constantly increasing, thus the amount of HCW. The capacity of the existing HCW disposal centers is insufficient, and the municipality is aiming at establishing a new HCWDCL. Healthcare and environmental experts face challenges in selecting the best location for the new disposal center among the alternatives. This study aims to provide a solid recommendation to decision-makers considering a comprehensive list of criteria by following the research steps presented in section “Methodology”.

### Identification of criteria and sub-criteria

After reviewing the literature and conducting in-depth interviews with experts, 40 effective sub-criteria were identified in the evaluation and prioritization of HCWDCL. The identified sub-criteria are categorized into five main criteria: economic, environmental, social, technical, and geological. The used criteria and sub-criteria and their symbols are presented in Table 3. As discussed in the literature review section, 35 sub-criteria are extracted from the previous research. Moreover, five new sub-criteria relevant to the case study are suggested by the experts. These new sub-criteria are “Distance from power lines”, “Burial fee”, “Project construction cost”, “Odor emission rate”, and “Lack of leachate control”.

**Table 3:** The used criteria and sub-criteria and their weights.

Main criteria	Weight of criteria	Sub-criteria	Weight of sub-criteria	Final weight	Cost or benefit
Economic (${c}_{1}$)	0.4822	Operating costs (C_11_)	0.2910	0.1404	−
		HW transportation cost to disposal facilities (C_12_)	0.2366	0.1141	−
		The cost of repairs and maintenance (C_13_)	0.1311	0.0632	−
		Investment cost (C_14_)	0.1178	0.0568	−
		Land price (C_15_)	0.0624	0.0301	−
		Burial fee (C_16_)	0.0724	0.0349	−
		Possibility of future expansion (C_17_)	0.0658	0.0317	+
		Project construction cost (C_18_)	0.0230	0.0111	−
Environmental (${c}_{2}$)	0.2449	Contamination (C_21_)	0.2709	0.0663	−
		Distance from surface water (C_22_)	0.2135	0.0523	+
		Noise pollution (C_23_)	0.1632	0.0400	−
		The effect of greenhouse gas emissions (C_24_)	0.1360	0.0333	−
		Depth of groundwater (C_25_)	0.0802	0.0196	+
		Distance from residential areas (C_26_)	0.0617	0.0151	+
		Odor emission rate (C_27_)	0.0473	0.0116	−
		Distance from power lines (C_28_)	0.0273	0.0067	+
Social (${c}_{3}$)	0.1583	Social acceptance (C_31_)	0.2297	0.0364	+
		Population density (C_32_)	0.2339	0.0370	−
		Archeological importance (C_33_)	0.2048	0.0324	−
		Distance from communities (C_34_)	0.0920	0.0146	+
		Health and safety (C_35_)	0.0940	0.0149	+
		Adherence to rules and regulations (C_36_)	0.0692	0.0110	+
		Land use (C_37_)	0.0328	0.0052	−
		Lack of municipal problems (C3_8_)	0.0437	0.0069	+
Technical (${c}_{4}$)	0.0762	Landfill capacity (C_41_)	0.3135	0.0239	+
		Availability of workforce (C_42_)	0.2021	0.0154	+
		Accessibility to the road (C_43_)	0.1292	0.0098	+
		Infrastructure development (C_44_)	0.1004	0.0077	+
		Distance to the garbage collection point (C_45_)	0.0898	0.0068	−
		Distance to a complex of waste sorting (C_46_)	0.0617	0.0047	−
		Distance from mines (C_47_)	0.0694	0.0053	+
		Lack of leachate control (C_48_)	0.0340	0.0026	−
Geological (${c}_{5}$)	0.0383	Soil type (C_51_)	0.2607	0.0100	−
		Height (C_52_)	0.1810	0.0069	−
		Distance from the fault (C_53_)	0.1623	0.0062	+
		Slope (C_54_)	0.1829	0.0070	−
		Wind problems (C_55_)	0.0790	0.0030	−
		Flooding (C_56_)	0.0644	0.0025	−
		Precipitation estimates (C_57_)	0.0427	0.0016	−
		Vegetation (C_58_)	0.0269	0.0010	−

### Determining the weight of criteria and sub-criteria

The AHP method is used to obtain the weights of the main criteria and their related sub-criteria.

Six decision matrices are employed to determine the weights of the used sub-criteria. The first pairwise comparisons matrix is a 5 × 5 structure that is used to contrast the main criteria and obtain their respective weights. The second through sixth pairwise comparison matrices are utilized to compare and obtain the weights of the sub-criteria related to each of the first through fifth main criteria, respectively. As each main criterion encompasses eight sub-criteria, the second through sixth pairwise comparison matrices are configured as an 8 × 8 structure. The final weight of each sub-criterion is calculated by multiplying its weight by the weight of its corresponding main criterion. The inconsistency rates of the first through sixth comparison matrices are 0.068, 0.077, 0.1, 0.056, 0.085, and 0.082, respectively.

Each pairwise comparison matrix is derived from the initial questionnaire. The geometric mean is used to consolidate these matrices from various experts into an integrated pairwise comparison matrix. Subsequent calculations are then performed on this integrated matrix. The final comparison matrixes can be found in Tables S1–S6 in the Supplementary Material.

The weight of the main criteria and sub-criteria are shown in Table 3. The ranking of the criteria based on their weight shows that the most important criteria are “economic” (0.4822), “environmental” (0.2450), and “social” (0.1583), respectively. Also, the sub-criteria of “operating cost” (0.1404), “transportation cost” (0.1141), and “pollution” (0.0664) have the most importance, respectively.

### Determining candidate alternatives for the HCW disposal center

According to the experts’ opinion, four distinct locations in the south (*L*_*1*_), southeast (*L*_*2*_), east (*L*_*3*_), and northeast (*L*_*4*_) of the province were identified and considered as alternatives for establishing a new HCW disposal center.

### Creating a decision matrix

The second questionnaire is used to collect the data required for the decision matrix. This matrix is used to determine the score of each alternative in each criterion. The final obtained decision matrix is presented in Table 4. The rank of each alternative in each sub-criterion when the decision matrix scores were defuzzified is presented in Fig. S2 in Supplementary Material.

**Table 4:** Decision matrix.

	L1	L2	L3	L4
$C_{11}$	(2.15, 2.75, 3.2)	(1.63, 2.38, 3.13)	(1.1, 1.63, 2.38)	(2.15, 2.75, 3.43)
$C_{12}$	(1.78, 2.38, 3.05)	(1.5, 2.25, 3)	(1, 1.38, 2.13)	(1.98, 2.5, 3.25)
$C_{13}$	(1.9, 2.5, 3.03)	(2.25, 3, 3.6)	(1.3, 1.75, 2.5)	(1.95, 2.63, 3.38)
$C_{14}$	(2.65, 3.25, 3.63)	(1.25, 2, 2.75)	(1.05, 1.5, 2.25)	(1.65, 2.25, 3)
$C_{15}$	(1.95, 2.63, 3.38)	(1.63, 2.38, 3.05)	(0.83, 1.13, 1.88)	(2.75, 3.5, 4.25)
$C_{16}$	(1.55, 2, 2.6)	(1.25, 2, 2.75)	(2.08, 2.75, 3.5)	(2.45, 3.13, 3.65)
$C_{17}$	(1.5, 1.88, 2.4)	(1.63, 2.38, 3.13)	(1.03, 1.63, 2.38)	(1.23, 1.75, 2.5)
$C_{18}$	(1.98, 2.5, 3.03)	(2, 2.75, 3.5)	(0.88, 1.25, 2)	(2.1, 2.63, 3.38)
$C_{21}$	(2.25, 3, 3.6)	(1.75, 2.5, 3.25)	(0.63, 1, 1.75)	(2.75, 3.5, 4.25)
$C_{22}$	(2, 2.75, 3.35)	(1, 1.75, 2.5)	(0.1, 0.25, 1)	(2.75, 3.5, 4.25)
$C_{23}$	(0.28, 0.5, 1.25)	(1.88, 2.63, 3.38)	(2.1, 2.63, 3.38)	(2.13, 2.88, 3.63)
$C_{24}$	(3.83, 4.5, 4.58)	(1.75, 2.5, 3.25)	(1.53, 2.13, 2.88)	(0.53, 0.75, 1.5)
$C_{25}$	(2.38, 3.13, 3.8)	(1.25, 2, 2.75)	(1.28, 1.88, 2.63)	(2.95, 3.63, 4.23)
$C_{26}$	(1.73, 2.25, 2.93)	(1.13, 1.88, 2.63)	(1.38, 2.13, 2.88)	(1.9, 2.5, 3.25)
$C_{27}$	(1.7, 2.38, 2.98)	(1.6, 2.13, 2.8)	(1.8, 2.25, 2.78)	(1.83, 2.5, 3.18)
$C_{28}$	(3.33, 4, 4.23)	(1.63, 2.38, 3.13)	(0.2, 0.5, 1.25)	(1.1, 1.63, 2.38)
$C_{31}$	(1.3, 1.75, 2.5)	(1.75, 2.5, 3.1)	(1.25, 1.63, 2.38)	(2.63, 3.38, 3.98)
$C_{32}$	(0.7, 1, 1.68)	(1.88, 2.63, 3.38)	(1.65, 2.25, 3)	(1.7, 2.38, 3.13)
$C_{33}$	(3.15, 3.75, 4.05)	(1.75, 2.5, 3.25)	(0.88, 1.25, 2)	(1.43, 1.88, 2.63)
$C_{34}$	(1.2, 1.88, 2.63)	(1.63, 2.38, 3.13)	(0.7, 1, 1.75)	(3, 3.75, 4.43)
$C_{35}$	(1.28, 1.5, 2.03)	(1.75, 2.5, 3.25)	(1.9, 2.5, 3.25)	(1.63, 2.38, 3.13)
$C_{36}$	(3.25, 4, 4.3)	(1.25, 2, 2.75)	(1.05, 1.5, 2.25)	(1.2, 1.5, 2.25)
$C_{37}$	(1.55, 2, 2.75)	(1.88, 2.63, 3.38)	(2.08, 2.75, 3.5)	(2.5, 3.25, 4)
$C_{38}$	(3.2, 3.88, 4.1)	(1.63, 2.38, 3.13)	(1.4, 2, 2.75)	(0.95, 1.25, 2)
$C_{41}$	(1.9, 2.5, 3.18)	(1.38, 2.13, 2.88)	(1.7, 2.38, 3.13)	(2.7, 3.38, 3.98)
$C_{42}$	(2.18, 2.63, 3.08)	(1.38, 2.13, 2.88)	(1.03, 1.63, 2.38)	(1.03, 1.63, 2.38)
$C_{43}$	(2.28, 2.88, 3.33)	(1.45, 2.13, 2.88)	(0.83, 1.13, 1.8)	(1.9, 2.5, 3.25)
$C_{44}$	(2.25, 3, 3.53)	(1.45, 2.13, 2.8)	(0.65, 0.88, 1.55)	(1.9, 2.5, 3.25)
$C_{45}$	(1.43, 1.88, 2.55)	(2.13, 2.88, 3.48)	(1.78, 2.38, 3.13)	(2.08, 2.75, 3.5)
$C_{46}$	(2.7, 3.38, 3.83)	(1.63, 2.38, 3.05)	(1.6, 2.13, 2.8)	(1.43, 1.88, 2.63)
$C_{47}$	(2.03, 2.63, 3.3)	(1.45, 2.13, 2.88)	(2.13, 2.88, 3.55)	(2.33, 3, 3.68)
$C_{48}$	(1.53, 2.13, 2.8)	(1.13, 1.88, 2.63)	(0.45, 0.75, 1.5)	(2.03, 2.63, 3.3)
$C_{51}$	(0.98, 1.5, 2.25)	(1.13, 1.88, 2.63)	(0.85, 1.38, 2.13)	(2.75, 3.5, 4.25)
$C_{52}$	(0.28, 0.5, 1.25)	(2.17, 2.92, 3.67)	(1.68, 2.13, 2.88)	(2.25, 3, 3.68)
$C_{53}$	(2.83, 3.5, 3.95)	(1.61, 2.36, 3.11)	(0.7, 1, 1.75)	(2.03, 2.63, 3.38)
$C_{54}$	(1.58, 2.25, 2.93)	(1.38, 2.13, 2.88)	(0.75, 1.13, 1.88)	(2.88, 3.63, 4.3)
$C_{55}$	(2.05, 2.5, 2.95)	(1.5, 2.25, 3)	(1.53, 2.13, 2.88)	(1.35, 1.88, 2.63)
$C_{56}$	(1.55, 2, 2.53)	(1.63, 2.38, 3.13)	(0.75, 1.13, 1.88)	(2.15, 2.75, 3.5)
$C_{57}$	(2.53, 3.13, 3.65)	(1.5, 2.25, 3)	(1.65, 2.25, 3)	(1.85, 2.38, 3.13)
$C_{58}$	(3.33, 4, 4.3)	(1.5, 2.25, 3)	(1.85, 2.38, 3.13)	(1.3, 1.75, 2.5)

### Ranking the HCWDCLs using the PROMSIS method

In this section, the results of the implementation of the PROMSIS method are presented.

The ranking proposed by the PROMSIS method is presented in Table 5. The results show that places ${L}_{4}$, ${L}_{3}$, ${L}_{1}$ and ${L}_{2}$ have the highest priority for establishing an HCW disposal center, respectively.

**Table 5:** Results of the PROMSIS method.

Alternative	$DPIS_{i}^{{}}$	$DNIS_{i}$	*Φ*_*i*_	$NDPIS_{i}$	$NDNIS_{i}$	*NΦ*_*i*_	$Q_{i}$	Rank
$L_{1}$	0.126	0.299	− 0.2341	0.3133	0.5544	− 0.2653	0.4799	3
$L_{2}$	0.153	0.272	− 0.2066	0.3805	0.5043	− 0.2342	0.4152	4
$L_{3}$	0.342	0.08	0.7606	0.8504	0.1483	0.8621	0.543	2
$L_{4}$	0.074	0.348	− 0.3199	0.184	0.6452	− 0.3626	0.6057	1

### Sensitivity analysis

In this section, we conduct three distinct analyses to evaluate the sensitivity of the fuzzy PROMSIS against variations in the criteria weight. The five primary criteria employed to appraise the candidate HCWDCLs are economic, environmental, social, technical, and geological.

The first analysis performs a sensitivity review by exploring different combinations of criteria weights. The second analysis calculates the ranking of alternatives based on each of the five criteria individually. The third analysis examines the effect of four criteria on ranking alternatives while excluding one of the criteria.

For the first analysis, six scenarios are considered to execute the sensitivity investigation. In the initial scenario, the weight of the criteria is considered the same as the ones derived through the AHP method. In the second scenario, the weight of the economic criterion is increased by 10%, and the outcome of the fuzzy PROMSIS method is assessed. Similarly, in the third, fourth, fifth, and sixth scenarios, the weight of the environmental, social, technical, and geological criteria is respectively increased by 10%.

The second and third analyses each comprise five scenarios. The second analysis determines the ranks of alternatives when only one of the five main criteria is considered individually. In the third analysis, the ranks of alternatives are calculated by considering the effect of four criteria and excluding one of the criteria.

**Figure 1:**
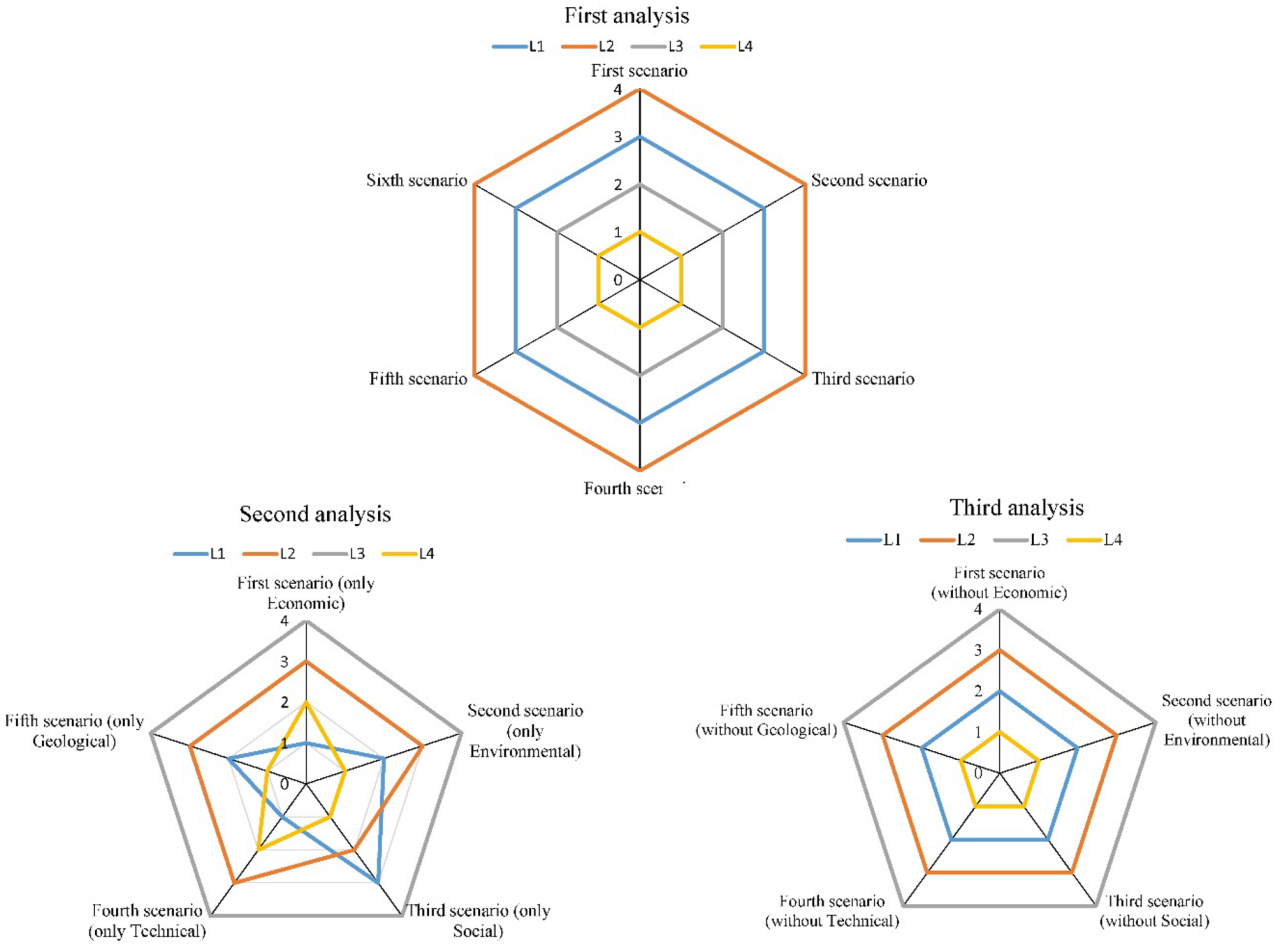
Sensitivity analysis.

Figure 1 presents the results of the sensitivity analysis. The findings from the first analysis indicate that the ranking of alternatives remains constant. This shows the low sensitivity of the PROMSIS to changes in the weight of the criteria within the ranges considered. The second analysis's results reveal that *L*_*3*_ has the lowest rank in all scenarios. Additionally, *L*_*4*_ achieves the highest rank in the second, third, and fifth scenarios and the second-highest rank in the first and fourth scenarios. The third analysis's results demonstrate that the ranking of alternatives remains consistent across various scenarios. Collectively, all analyses demonstrate the reliability of the PROMSIS method, as it is not overly sensitive to the input parameters and provides reasonably consistent results.

### Comparison of fuzzy PROMSIS with other methods

Since the PROMSIS originated from TOPSIS and PROMETHEE methods, the results obtained by the fuzzy PROMSIS method are compared with the results of the fuzzy PROMETHEE^37^ and fuzzy TOPSIS^38,39^ for the case study. Table 6 shows the obtained results after implementing these three methods. Moreover, the obtained results by the fuzzy WASPAS method^40^ and the fuzzy TOPKOR method^41^ are mentioned in Table 6.

**Table 6:** Comparison between results of the PROMSIS, TOPSIS, and PROMETHEE methods.

Alternative	Fuzzy TOPSIS		Fuzzy PROMETHEE		Fuzzy PROMSIS		fuzzy WASPAS	fuzzy TOPKOR
	*CC*_*i*_	Rank	Φ(a)	Rank	*Q*_*i*_	Rank	Rank	Rank
*L*_*1*_	0.703	2	-0.2341	3	0.4799	3	2	4
*L*_*2*_	0.641	3	-0.2066	2	0.4152	4	4	3
*L*_*3*_	0.19	4	0.7606	1	0.543	2	1	1
*L*_*4*_	0.826	1	-0.3199	4	0.6057	1	3	2

The results show that because of different viewpoints of the compared MCDM methods, the ranking provided by the considered methods is not the same. In the fuzzy PROMETHEE method, the alternatives $L_{3}$, $L_{2}$, $L_{1}$ and ${ }L_{4}$ have the highest priority, respectively. While in the fuzzy TOPSIS method, the alternatives $L_{4}$, $L_{1}$, $L_{2}$ and ${ }L_{3}$ have the highest priority, respectively.

To compare of the performance of fuzzy PROMSIS method against fuzzy TOPSIS and Fuzzy PROMETHEE methods, an index named the Sum of Differences in Ranking the Alternatives (SDRA) index is used. If *A* and *B* are two different rankings of alternatives, the *SDRA*(*A*,*B*) index shows the amount of difference in *A* and *B* ranking, and it is defined as Eq. (42).

42 $$SDRA(A,B) = \sum\limits_{i = 1}^{m} {\left| {{\text{Rank}}\,{\text{of}}\,i{{{\text{th}}}} \,{\text{alternative}}\,{\text{in}}\,A\, - {\text{Rank}}\,{\text{of}}\,i{{{\text{th}}}} \,{\text{alternative}}\,{\text{in}}\,B} \right|}$$

The lower value of this index means that these two rankings verify each other. Table 7 shows that the sum of the *SDRA* index for the fuzzy PROMSIS method is lower than the other methods. This result indicates that the ranking proposed by the fuzzy PROMSIS has less deviation as compared with other methods.

**Table 7:** Comparing the amount of difference in the alternatives’ ranking.

*SDRA*	Fuzzy PROMSIS	Fuzzy PROMETHEE	Fuzzy TOPSIS	Fuzzy WASPAS	Fuzzy TOPKOR	Sum of *SDRA*
Fuzzy PROMSIS	0	6	4	4	4	18
Fuzzy PROMETHEE	6	0	8	4	4	22
Fuzzy TOPSIS	4	8	0	6	6	24

### Discussion

The results show that the sub-criteria “operating cost” and “transportation cost” have the most importance, respectively. Although the investment cost accounted for a large amount of the budget, it obtained a lower weight. It could be interpreted that the operation and transportation costs are repeated daily, and their cumulative amount will be significant in the long run.

The proposed PROMSIS method tries to integrate the viewpoints of the PROMETHEE and TOPSIS methods. PROMETHEE method gives a higher priority to an alternative with a higher value of net preference flow value. On the other hand, the TOPSIS method gives a higher priority to alternatives with a lower distance from the positive ideal solution (PIS) and a higher distance from the negative ideal solution (NIS). PROMSIS method integrates these viewpoints and gives a higher priority to an alternative with a low distance from PIS, a high distance from NIS, and a high value for net preference. Considering more viewpoints in the decision-making process increases its results' reliability.

Comparing the results of fuzzy PROMSIS and fuzzy PROMETHEE methods show a difference in ranking the alternatives. The highest priority is given to $L_{3}$ by PROMETHEE while $L_{4}$ received the first rank by PROMSIS. This difference has ruts in the viewpoints of the two methods. Using the PROMETHEE method, $L_{3}$ has the highest value of the input preference flow and the lowest value of the output preference flow. Thus, it obtains the best rank by the fuzzy PROMETHEE method. Applying the PROMSIS method, although $L_{3}$ has a better net preference flow (0.7606) than $L_{4}$ (− 0.3199), but the closeness coefficient of $L_{4}$ (0.826) is better than $L_{3}$ (0.19). Therefore, the fuzzy PROMSIS index for $L_{4}$ is better than $L_{3}$, and receives a higher rank.

Comparing the results of implementing fuzzy PROMSIS and fuzzy TOPSIS methods show that $L_{4}$ received the highest priority by both methods. However, there is a difference in the ranking of other alternatives. For instance, the results show that the closeness coefficient of $L_{2}$ is better than $L_{3}$ and therefore, its obtained a higher rank by the TOPSIS method. But the net preference flow of $L_{3}$ is better than $L_{2}$ thus, in the ranking provided by the fuzzy PROMSIS, $L_{3}$ has higher priority than $L_{2} .$

## Conclusion and scopes of future research

This research presented a new hybrid MCDM method for evaluating and ranking HCWDCL, named PROMSIS. The study benefited from fuzzy theory for describing the uncertainty of the problem parameters. The PROMSIS was used to rank the alternative HCWDCLs in a case study. First, the related criteria and sub-criteria for evaluating HCWDCLs were identified. Then the weight of the considered criteria and sub-criteria were determined using the AHP method. After determining the score of each candidate site in each criterion, they were ranked by the fuzzy PROMSIS method. In this research, 40 sub-criteria were used for evaluating and prioritizing the alternative HCWDCLs. 35 of the sub-criteria were identified through a literature review, and five new sub-criteria relevant to the case study were suggested by the experts. The new sub-criteria are “Distance from power lines”, “Burial fee”, “Project construction cost”, “Odor emission rate”, and “Lack of leachate control”. All the sub-criteria are categorized into five major criteria: economic, environmental, social, technical, and geological. The results of the study suggested that alternative four is the best location for establishing a new HCW disposal center. The results analysis also indicated that economic, environmental, and social aspects were the most important criteria for ranking the alternatives in the studied case, respectively. Moreover, the operating cost, transportation cost, and pollution were identified as the most influential sub-criteria in the decision-making process.

The findings of this study are closely related to the data of the considered case study as well as the expert opinions, their scores in pairwise comparisons, and decision matrix questionnaires. Therefore, the results can be altered by variations in expert opinions. Moreover, the potential HCWDCLs evaluated in this research may differ from those in other studies. Consequently, the number of candidate locations and their evaluations across various criteria could differ from those considered in this study.

Although the methodology proposed in this study is adaptable, the results could vary depending on the dataset utilized (e.g., different geographic, demographic, and economic characteristics of the locations). Therefore, while the study provides a valuable framework for similar analyses, the results cannot be generalized. Furthermore, the results of the study might change over time with changes to economic fluctuations, population growth, advancements in healthcare waste treatment technology, etc. However, the identified criteria and sub-criteria provide the decision-makers in recycling disposal and healthcare centers with a comprehensive view of the important factors to be considered when choosing a disposal center. Moreover, the proposed fuzzy PROMSIS method could be used by experts as decision support when deciding on the HCWDCL.

Future studies can benefit from the proposed PROMSIS to deal with other cases and evaluate its performance against other MCDM methods. Moreover, identifying other criteria for selecting the best HCWDCL could be considered a field for future research. Using other MCDM methods for determining the weight of criteria and combing it with the proposed PROMSIS could also be a promising extension of the current study.

### Supplementary Information

Supplementary Information.

## Data Availability

The data supporting this study's findings are available from the corresponding author upon reasonable request.
