# Supplementary material for: Evaluating and prioritizing the healthcare waste disposal center locations using a hybrid multi-criteria decision-making method
Source: Sci Rep. 2023 Sep 13;13:15130. doi: 10.1038/s41598-023-42455-w (PMC10499883; doi:10.1038/s41598-023-42455-w)
Supplement: Supplementary file 1 — Supplementary Information. [file 41598_2023_42455_MOESM1_ESM.docx]

**Supplementary Material**


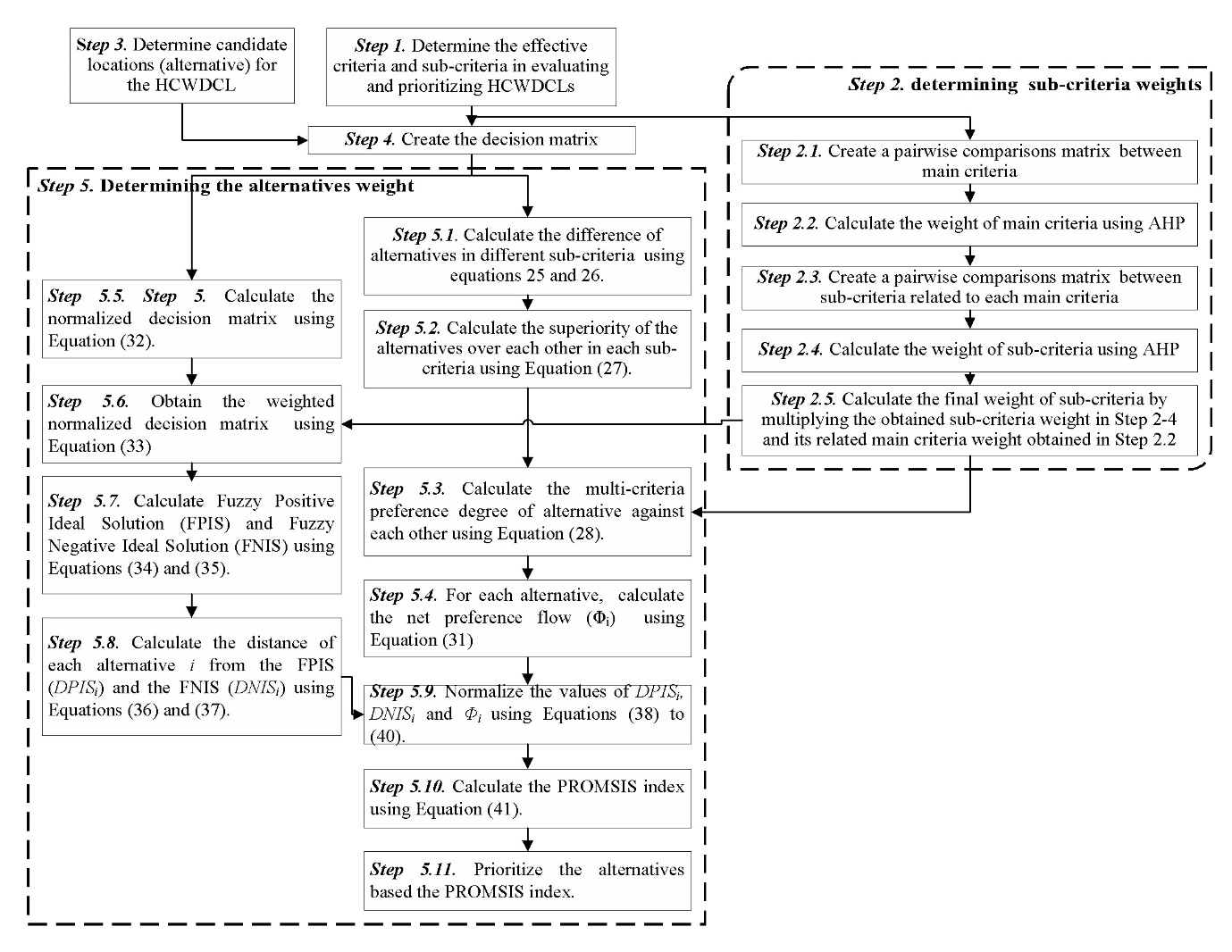


**Figure S1.** Research methodology steps


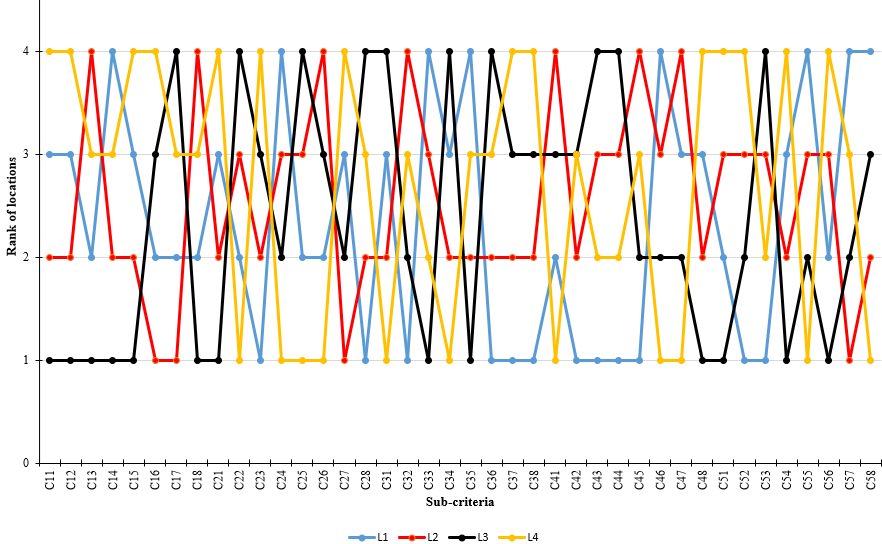


**Figure S2.** Ranks of alternatives in each sub-criterion

**Table S1.** Pairwise comparisons matrix between main criteria

|  | C_1_ | C_2_ | C_3_ | C_4_ | C_5_ |
| --- | --- | --- | --- | --- | --- |
| C_1_ | 1 | 3.081397 | 3.968014 | 7.008117 | 6.459362 |
| C_2_ | 0.324528 | 1 | 2.345541 | 3.833738 | 7.062261 |
| C_3_ | 0.252015 | 0.426341 | 1 | 2.159439 | 7.300372 |
| C_4_ | 0.142692 | 0.260842 | 0.463083 | 1 | 3.068716 |
| C_5_ | 0.154814 | 0.141598 | 0.136979 | 0.325869 | 1 |

**Table S2.** Pairwise comparisons matrix between economic sub-criteria

|  | C_11_ | C_12_ | C_13_ | C_14_ | C_15_ | C_16_ | C_17_ | C_18_ |
| --- | --- | --- | --- | --- | --- | --- | --- | --- |
| C_11_ | 1 | 2.663957 | 3.211329 | 2.330452 | 3.053907 | 3.702288 | 5.432689 | 8.027383 |
| C_12_ | 0.375381 | 1 | 1.244739 | 4.917496 | 5.238158 | 3.015676 | 5.061018 | 9 |
| C_13_ | 0.311398 | 0.803381 | 1 | 1.328643 | 1.374109 | 3.691873 | 3.18529 | 2.663957 |
| C_14_ | 0.429101 | 0.203356 | 0.752648 | 1 | 2.666081 | 2.101507 | 2.491462 | 6.487794 |
| C_15_ | 0.327449 | 0.190907 | 0.727744 | 0.375082 | 1 | 0.432964 | 0.908003 | 4.068999 |
| C_16_ | 0.270103 | 0.331601 | 0.270865 | 0.475849 | 2.309662 | 1 | 0.966482 | 4.24612 |
| C_17_ | 0.184071 | 0.197589 | 0.313943 | 0.401371 | 1.101318 | 1.03468 | 1 | 7.183273 |
| C_18_ | 0.124574 | 0.111111 | 0.375381 | 0.154136 | 0.245761 | 0.235509 | 0.139212 | 1 |

**Table S3.** Pairwise comparisons matrix between environmental sub-criteria

|  | C_21_ | C_22_ | C_23_ | C_24_ | C_25_ | C_26_ | C_27_ | C_28_ |
| --- | --- | --- | --- | --- | --- | --- | --- | --- |
| C_21_ | 1 | 1.151021 | 1.472733 | 4.160817 | 6.06582 | 2.751634 | 6.575749 | 7.489813 |
| C_22_ | 0.868794 | 1 | 1.341671 | 3.379634 | 1.680843 | 3.989198 | 7.590496 | 4.883574 |
| C_23_ | 0.67901 | 0.745339 | 1 | 0.633538 | 1.993711 | 5.891598 | 5.341168 | 3.607445 |
| C_24_ | 0.240337 | 0.29589 | 1.578437 | 1 | 1.297279 | 3.0219 | 5.326143 | 6.861987 |
| C_25_ | 0.164858 | 0.594939 | 0.501577 | 0.770844 | 1 | 1.028728 | 2.951546 | 3.167321 |
| C_26_ | 0.36342 | 0.250677 | 0.169733 | 0.330918 | 0.972075 | 1 | 2.72963 | 2.392462 |
| C_27_ | 0.152074 | 0.131744 | 0.187225 | 0.187753 | 0.338805 | 0.36635 | 1 | 6.599707 |
| C_28_ | 0.133515 | 0.204768 | 0.277204 | 0.14573 | 0.315724 | 0.417979 | 0.151522 | 1 |

**Table S4.** Pairwise comparisons matrix between social sub-criteria

|  | C_31_ | C_32_ | C_33_ | C_34_ | C_35_ | C_36_ | C_37_ | C_38_ |
| --- | --- | --- | --- | --- | --- | --- | --- | --- |
| C_31_ | 1 | 0.75417 | 0.736039 | 7.790057 | 1.914489 | 5.250596 | 5.867975 | 3.044177 |
| C_32_ | 1.325962 | 1 | 0.927031 | 3.136565 | 2.726241 | 2.688881 | 7.50496 | 6.861212 |
| C_33_ | 1.358624 | 1.078713 | 1 | 2.91001 | 3.15331 | 2.360728 | 3.956851 | 3.151905 |
| C_34_ | 0.128369 | 0.31882 | 0.343641 | 1 | 0.779977 | 1.383622 | 6.03429 | 3.151905 |
| C_35_ | 0.522332 | 0.366805 | 0.317127 | 1.282089 | 1 | 2.095596 | 2.293782 | 2.513889 |
| C_36_ | 0.190455 | 0.371902 | 0.423598 | 0.722741 | 0.477191 | 1 | 2.839053 | 1.939361 |
| C_37_ | 0.170417 | 0.133245 | 0.252726 | 0.16572 | 0.435961 | 0.35223 | 1 | 0.745091 |
| C_38_ | 0.328496 | 0.145747 | 0.317268 | 0.317268 | 0.39779 | 0.515634 | 1.342118 | 1 |

**Table S5.** Pairwise comparisons matrix between technical sub-criteria

|  | C_41_ | C_42_ | C_43_ | C_44_ | C_45_ | C_46_ | C_47_ | C_48_ |
| --- | --- | --- | --- | --- | --- | --- | --- | --- |
| C_41_ | 1 | 1.513331 | 2.294803 | 3.545174 | 4.643984 | 6.979567 | 6.13076 | 6.645675 |
| C_42_ | 0.660794 | 1 | 1.426944 | 2.066105 | 2.730538 | 4.95328 | 4.563717 | 3.972946 |
| C_43_ | 0.435767 | 0.700799 | 1 | 1.048122 | 1.876028 | 2.550849 | 3.143039 | 2.726241 |
| C_44_ | 0.282074 | 0.484003 | 0.954087 | 1 | 1.311019 | 3.007192 | 1.370243 | 2.140174 |
| C_45_ | 0.215332 | 0.366228 | 0.533041 | 0.762765 | 1 | 1.655132 | 3.202295 | 3.678471 |
| C_46_ | 0.143275 | 0.201886 | 0.392026 | 0.332536 | 0.604182 | 1 | 2.370385 | 3.574155 |
| C_47_ | 0.163112 | 0.21912 | 0.318163 | 0.729797 | 0.312276 | 0.421872 | 1 | 7.825228 |
| C_48_ | 0.150474 | 0.251702 | 0.366805 | 0.467252 | 0.271852 | 0.279786 | 0.127792 | 1 |

**Table S6.** Pairwise comparisons matrix between geological sub-criteria

|  | C_51_ | C_52_ | C_53_ | C_54_ | C_55_ | C_56_ | C_57_ | C_58_ |
| --- | --- | --- | --- | --- | --- | --- | --- | --- |
| C_51_ | 1 | 1.231144 | 3.131629 | 2.008181 | 4.122326 | 4.63092 | 5.664525 | 5.591248 |
| C_52_ | 0.812252 | 1 | 2.653105 | 1.121582 | 2.825235 | 2.986516 | 2.653105 | 3.752057 |
| C_53_ | 0.319323 | 0.376917 | 1 | 0.418972 | 2.730538 | 6.557524 | 6.66961 | 6.740533 |
| C_54_ | 0.497963 | 0.891598 | 2.386795 | 1 | 2.069361 | 2.130795 | 6.222966 | 9 |
| C_55_ | 0.242581 | 0.353953 | 0.366228 | 0.483241 | 1 | 0.592044 | 3.026663 | 5.903759 |
| C_56_ | 0.21594 | 0.334838 | 0.152497 | 0.469308 | 1.689064 | 1 | 1.597138 | 2.320958 |
| C_57_ | 0.176537 | 0.376917 | 0.149934 | 0.160695 | 0.330397 | 0.62612 | 1 | 2.8877 |
| C_58_ | 0.178851 | 0.266521 | 0.148356 | 0.111111 | 0.169384 | 0.430857 | 0.346296 | 1 |
